# A model-informed approach to assess the risk of immune checkpoint inhibitor-induced autoimmune myocarditis

**DOI:** 10.3389/fphar.2022.966180

**Published:** 2022-09-26

**Authors:** Solveig A. van der Vegt, Ying-Jie Wang, Liudmila Polonchuk, Ken Wang, Sarah L. Waters, Ruth E. Baker

**Affiliations:** ^1^ Wolfson Centre for Mathematical Biology, Mathematical Institute, University of Oxford, Oxford, United Kingdom; ^2^ Department of Cardiovascular Medicine, Radcliffe Department of Medicine, Wellcome Centre of Human Genetics, University of Oxford, Oxford, United Kingdom; ^3^ Pharmaceutical Research and Early Development, Roche Innovation Center Basel, F. Hoffmann-La Roche Ltd., Basel, Switzerland; ^4^ Oxford Centre for Industrial and Applied Mathematics, Mathematical Institute, University of Oxford, Oxford, United Kingdom

**Keywords:** myocarditis, autoimmunity, mathematical modelling, immune checkpoint inhibitors, T cells

## Abstract

Immune checkpoint inhibitors (ICIs), as a novel immunotherapy, are designed to modulate the immune system to attack malignancies. Despite their promising benefits, immune-related adverse events (IRAEs) may occur, and incidences are bound to increase with surging demand of this class of drugs in treating cancer. Myocarditis, although rare compared to other IRAEs, has a significantly higher fatal frequency. Due to the overwhelming complexity of the immune system, this condition is not well understood, despite the significant research efforts devoted to it. To better understand the development and progression of autoimmune myocarditis and the roles of ICIs therein, we suggest a new approach: mathematical modelling. Mathematical modelling of myocarditis has enormous potential to determine which parts of the immune system are critical to the development and progression of the disease, and therefore warrant further investigation. We provide the immunological background needed to develop a mathematical model of this disease and review relevant existing models of immunology that serve as the mathematical inspiration needed to develop this field.

## 1 Introduction

As patient survival increases for many forms of cancer, the long-term effects of both cancer itself and anti-cancer therapies, including immune checkpoint inhibitors (ICIs), are becoming of greater importance (Jain et al., 2017). Autoimmune myocarditis, inflammation of cardiac muscle tissue, is a rare but underdiagnosed disease with potentially fatal outcomes. Current data indicates that autoimmune myocarditis occurs in approximately 0.04–1.4% of cancer patients treated with ICIs, a treatment option for which over 40% of US cancer patients are eligible (Błyszczuk, 2019; Haslam and Prasad, 2019; Zhang et al., 2019; Palaskas et al., 2020). However, these figures are likely underestimates of the actual incidence rate of myocarditis as definitive diagnosis remains difficult (Zhang et al., 2016).

Although the reported incidence rate is low, the relatively high fatality rate of autoimmune myocarditis compared to other IRAEs, standing at 25–50%, warrants concern (Salem et al., 2018; Michel et al., 2019; Zhang et al., 2019). Despite a significant amount of experimental work being undertaken to understand this disease, much still remains unknown (Lv et al., 2011; Błyszczuk, 2019; Gil-Cruz et al., 2019). The inherent complexity of the immune system makes it difficult to determine the major pathways and triggers critical to the development and progression of myocarditis, and the roles that ICIs might play in these processes. Mathematical modelling can help in making these determinations. It provides a framework for understanding how multiple highly complex and nonlinear processes combine to produce the pathology observed in patients. Using mathematics to increase understanding of pathological processes has led to significant progress in other contexts, such as cancer (Dritschel et al., 2018; Brady and Enderling, 2019; Mahlbacher et al., 2019), neurological disorders (Hao and Friedman, 2016; Bertsch et al., 2017; Carbonell et al., 2018), and cystic fibrosis (Sandefur et al., 2017; Anagnostopoulou et al., 2019) to name but a few. In a system as overwhelmingly complex as the human immune system, making a distinction between critical and secondary processes in the development and progression of autoimmune myocarditis will be essential in moving research forward efficiently.

In this review, we discuss the current understanding of the causes (Section 2.1) and pathways (Section 2.2–2.4) involved in the development and progression of autoimmune myocarditis, and how these are affected by the presence of ICIs. This will pave the way for the development of mathematical models that can be used to identify the most critical cell types and interactions involved in this pathology. The model response to different perturbations, including model behaviour after the influx of different cell types could help to determine patient-specific maximum-tolerated ICI doses, taking into account specific patient risks factors of developing autoimmune myocarditis. In addition, the model predictions could be used to guide drug candidate selection in an *in vitro* assay, where the dosage of ICIs and the number of specific cells added to the system can be controlled. A predictive modelling approach is, therefore, a welcome first step towards determining treatment regimens that balance the benefits of treating cancer with the risk of developing autoimmune myocarditis. Ultimately, this research should help to improve the risk-benefit profile and guide clinical mitigation strategies by predicting what dose levels and regimens of ICIs a patient can receive without developing autoimmune myocarditis, or how patients with different characteristics might respond to the same treatment protocol.

Although various factors can cause myocarditis, it is generally believed that a transition to T-cell mediated autoimmunity eventually takes place, and we therefore focus on these pathways in our discussion of adaptive immunity (Section 2.3) (Bracamonte-Baran and Cihakova, 2017; Postow et al., 2018). As a starting point for a mathematical model of autoimmune myocarditis, we summarize the relevant immunology in a network graph containing the main cell types and interactions discussed in Section 2 (see Figure 3). We also highlight several existing mathematical models of the (auto)immune response or parts thereof (Section 3). Although none of them are sufficient on their own to describe the specific case of autoimmune myocarditis, they provide inspiration for what a model of this disease might look like. We conclude by highlighting the possible avenues of research into autoimmune myocarditis and the effects of ICIs that a mathematical model could contribute to. Frequently used abbreviations in this article are listed in Table 1.

**TABLE 1:** Frequently used abbreviations.

Abbreviation	Meaning
ICI	Immune checkpoint inhibitor
APC	Antigen-presenting
DC	Dendritic cell
(i/n)Treg cell	(induced/natural) T regulatory cell
Th cell	T helper cell
TCR	T cell receptor
PMN	Polymorphonuclear leukocyte
M1/M2	Macrophages of phenotype 1/2
MyHC*α*	Myosin heavy chain *α*
PD-(L)1	Programmed death-(ligand) 1
CTLA4	Cytotoxic T-lymphocyte-associated protein 4
MHC	Major histocompatibility complex
IFN	Interferon
IL	Interleukin
TGF*β*	Transforming growth factor *β*
NET	Neutrophil extracellular trap
ECM	Extracellular matrix
ODE	Ordinary differential equation
PDE	Partial differential equation

## 2 Immunology

**FIGURE 1:**
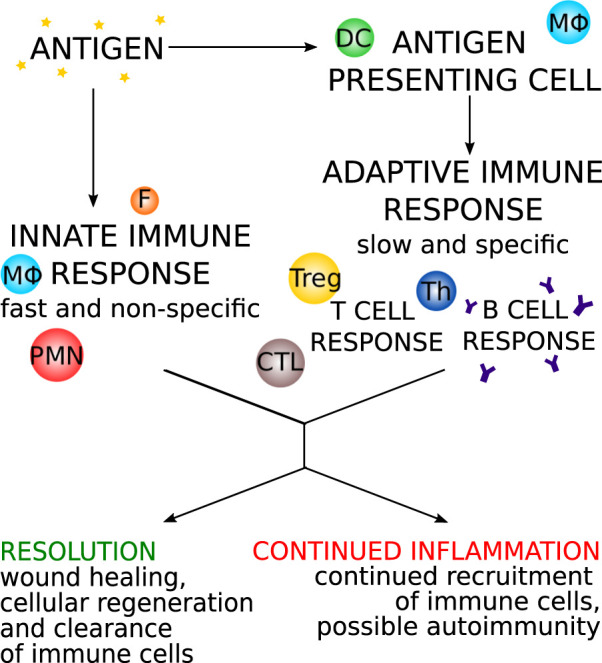
An overview of the progression of the immune response with two main arms: the innate response and the adaptive response. DC: dendritic cells, MΦ: macrophages/monocytes, PMN: polymorphonuclear leukocytes, F: fibroblast, Th: T helper cell, Treg: T regulatory cell, CTL: cytotoxic T cell.

The immune response is a powerful defense mechanism that, through its different pathways, launches a quick and specific attack against foreign pathogens. In autoimmune myocarditis, the full force of the immune system is directed against a self-antigen, a molecule that is a natural part of the body and to which the immune system is normally tolerant. This can have potentially lethal consequences (Lv et al., 2011; Błyszczuk, 2019). Below, we discuss the different causes of autoimmune myocarditis, as well as the development and possible outcomes of this disease. Figure 1 gives a broad overview of the immune response.

### 2.1 Causes

The antigens stimulating the autoimmune response in myocarditis can be released through a number of processes, including infection and spontaneous or drug-induced cell damage. Whatever the initial aetiology, myocarditis will, if unresolved, transition to a T-cell mediated autoimmune disease (Błyszczuk, 2019). There are several factors that can predispose an individual to developing autoimmune myocarditis, including certain human leukocyte antigen genes, being male, and deficiency in thymic training or T regulatory (Treg) cell induction (Bracamonte-Baran and Cihakova, 2017).

One of the main proteins believed to be a self-antigen targeted in autoimmune myocarditis is myosin heavy-chain *α* (MyHC*α*) (Lv et al., 2011; Błyszczuk, 2019). This intracellular protein, although not abundant, is unique to cardiomyocytes and is what is known as a cryptic antigen, meaning it is not or very little expressed in the thymus or peripheral lymphoid stromal cells (Rock and Kono, 2008; Lv et al., 2011). Peripheral lymphoid stromal cells are important for mediating peripheral tolerance (Lv et al., 2011). The thymus is a lymphoid organ where T cells mature, and which provides central tolerance. Presentation of a molecule in the thymus is required for the immune system to recognize it as “self” and tolerate it, i.e., not respond to it. As MyHC*α* is only present at very low levels in the thymus, T cells with TCRs specific to this antigen are not completely removed and MyHC*α*-specific T cells are known to be in circulation in humans (Lichtman, 2013; Błyszczuk, 2019; Gil-Cruz et al., 2019). This can lead to problems in several ways. For example, certain proteins of the commensal gut bacterial species *Bacteroides* mimic MyHC*α*, and an immune reaction against these bacteria can lead to severe autoimmune myocarditis when a second inflammatory trigger exposes the MyHC*α* in cardiac tissue (Gil-Cruz et al., 2019). Similarly, it has been found in certain patients that the T cells mobilized by cancer treatment are specific to an antigen that is present in the tumour as well as the heart and that this cross-reaction might constitute one pathway via which ICIs induce myocarditis (Postow et al., 2018). Additionally, experiments have shown that knocking out the gene for programmed cell death protein 1 (PD-1) leads to increasing levels of autoantibodies against cardiac antigens, and that knocking out the gene for cytotoxic T-lymphocyte-associated protein 4 (CTLA4) causes increased T cell infiltration across a number of organs, including the heart (Okazaki et al., 2003; Zhang et al., 2019). Both PD-1 and CTLA4 are involved in key regulatory pathways that control the immune response and prevent autoimmunity. They are also targets of ICIs; for example, they are blocked by nivolumab and ipilimumab, both of which have been shown to have autoimmune myocarditis as a possible side effect (Johnson et al., 2016).

Thus, several potential triggers, including ICIs, genetic characteristics and molecular mimicry, can increase the likelihood of an autoimmune reaction against cryptic cardiac antigens such as MyHC*α*, and thus the development of myocarditis.

### 2.2 Innate immunity

The immune response can be broadly divided into two pathways: the fast, non-specific innate immune response and the slower, pathogen-specific adaptive immune response. The former (also known as the acute inflammatory response) relies mainly on tissue-resident immune cells, and recruited polymorphonuclear leukocytes (PMNs) and macrophages, whereas the latter (also known as chronic inflammation) is executed by activated T cells and B cells that provide antigen-specific defence and memory functions (Serhan et al., 2010; Italiani and Boraschi, 2014).

The innate immune response is instrumental in the first few hours and days after infection or injury. It is activated in response to cues of tissue injury, or recognition of conserved motifs of foreign pathogens (Alberts et al., 2002; Serhan et al., 2010). A major pathway in the innate immune response is the complement cascade which produces both cytokines that attract PMNs, and opsonic and lytic factors that lead to the destruction of invading pathogens (Serhan et al., 2010). Macrophages play an important role in mediating the innate immune response, clearing pathogens and resolving inflammation (Bracamonte-Baran and Cihakova, 2017). Activated resident fibroblasts are important for maintaining tissue integrity and wound healing (Shinde and Frangogiannis, 2017). Below we discuss the roles of macrophages, PMNs, and fibroblasts further.

#### 2.2.1 Macrophages

Macrophages are among the most abundant cell types to accumulate in cardiac tissue in autoimmune myocarditis (Chow et al., 1989). Tissue-resident cardiac macrophages originate from embryonic cells and, during homeostasis, are primarily maintained through local proliferation (Epelman et al., 2014). Furthermore, throughout life cells of the monocyte-macrophage lineage are created in the bone marrow and released into the blood stream as monocytes, which can infiltrate cardiac tissue during inflammation (Serhan et al., 2010; Epelman et al., 2014; Shapouri-Moghaddam et al., 2018). When an invading pathogen or excessive cell death causes the release of pathogen-associated or damage-associated molecular patterns, tissue-resident macrophages act as sentinel cells. They initiate the recruitment of PMNs such as neutrophils by increasing their chemokine output, thus promoting the development of the innate immune response (Kolaczkowska and Kubes, 2013). During inflammation, tissue macrophages expand through proliferation and infiltrating macrophages are recruited from the blood stream and activated by T helper (Th) cells (Janeway et al., 2001; Epelman et al., 2014).

Activated macrophages sit on a phenotypic spectrum between M1 and M2, which are pro- and anti-inflammatory phenotypes, respectively. The M1 phenotype is induced by Th1-secreted cytokines such as IFN*γ* and tumour necrosis factor *α*. Differentiation towards the M2 phenotype is stimulated by the Th2-derived cytokines interleukin (IL)-4 and IL-13, as well as IL-10, TGF*β* and other anti-inflammatory compounds (Italiani and Boraschi, 2014; Shapouri-Moghaddam et al., 2018). Macrophages retain a high level of phenotypic plasticity over their life time, allowing them to respond to environmental cues. Their origin somewhat steers their behaviour, however, with tissue-resident macrophages maintaining an essential cardioprotective role, and infiltrating macrophages being important for coordinating inflammation while being less involved in antigen sampling and removal of dead cardiomyocytes (Epelman et al., 2014; Dick et al., 2019).

Macrophages have three main functions: 1) phagocytosis of pathogens, dead or infected cells and cell debris; 2) antigen presentation; and 3) secretion of immune-modulating cytokines (Shapouri-Moghaddam et al., 2018). The level to which macrophages perform each of these functions depends on where on the phenotypic spectrum they sit. M1 macrophages stimulate the Th1 cell-mediated immune response by secreting IL-12, IL-15 and IL-18, and have great phagocytic and antigen-presenting abilities. M2 macrophages modulate the Th2 response, are involved in tissue remodeling and wound healing, and immune tolerance, and they generally decrease the number of active T cells (Serhan et al., 2010; Bracamonte-Baran and Cihakova, 2017; Yang et al., 2017). They secrete TGF-*β* and IL-10, both anti-inflammatory cytokines. A proper balance between the two phenotypes is essential for an effective immune response (Atri et al., 2018).

#### 2.2.2 Polymorphonuclear leukocytes

Polymorphonuclear leukocytes are a group of immune cells that include neutrophils, eosinophils, basophils and mast cells (Serhan et al., 2010). Neutrophils and eosinophils in particular have been implicated in myocarditis (Diny et al., 2017; Weckbach et al., 2019).

Neutrophils phagocytose pathogens, after which granules containing highly toxic reactive oxygen species merge with the vacuole containing the pathogen. This process can contribute to inflammation when the contents of the granules leak out and end up on normal tissue. Another major effector pathway for neutrophils is through neutrophil extracellular traps (NETs). NETs are a scaffold of decondensed (nuclear and mitochondrial) chromatin loaded with cytosolic and granule proteins. They trap and kill most kinds of foreign pathogens and inhibit the dissemination of some. However, NETs are known to lead to tissue injury and play a role in immune-mediated diseases, including myocarditis (Mayadas et al., 2014; Papayannopoulos, 2018; Weckbach et al., 2019). Furthermore, neutrophils can release numerous toxic compounds, such as reactive oxygen species, that can damage cardiac tissue and even induce apoptosis in cardiomyocytes (Fulda et al., 2010; Németh and Mócsai, 2012). Lastly, neutrophils provide positive feedback by stimulating the maturation and/or activation of macrophages, dendritic cells (DCs), Th cells and natural killer cells, amongst others (Kaplan, 2013).

Eosinophils can likewise degranulate to attack foreign pathogens (Diny et al., 2017). They are also heavily involved in regulating both the innate and adaptive immune responses through the secretion of multiple cytokines with immune-modulating functions. They can, for example, stimulate the differentiation and recruitment of Th2 cells, and act as APCs (Wen and Rothenberg, 2016).

Both neutrophils and eosinophils can cause significant damage to the healthy tissue surrounding the inflamed site (Németh and Mócsai, 2012; Diny et al., 2017). Because cardiomyocytes have little to no regenerative capabilities, this can have severe consequences for heart function as damaged cells will be replaced by stiff collagenous fibres and scar tissue will develop (Serhan et al., 2010; Heymans et al., 2016).

#### 2.2.3 Fibroblasts

The microenvironment of cells contains extracellular matrix (ECM) proteins, and blood and lymph vessels, but is primarily defined by stromal cells. The most common type of stromal cells are fibroblasts, which play an important role in maintaining and modulating the ECM, and in the production of cytokines to recruit other inflammatory cells (Souders et al., 2009; Serhan et al., 2010). For example, IL-17 induces the production and excretion of myeloid cytokines, particularly granulocyte-macrophage colony-stimulating factor, in cardiac fibroblasts. These cytokines recruit neutrophils and induce a pro-inflammatory, pro-fibrotic phenotype in macrophages (Wu et al., 2014; Hou et al., 2019).

In myocarditis, cardiac resident fibroblasts are activated through damage-associated molecular patterns released by damaged or dead cardiomyocytes, and by macrophages through the cytokines TGF-*β* and tumour necrosis factor *α* (Bracamonte-Baran and Cihakova, 2017; Shinde and Frangogiannis, 2017; Hou et al., 2019; Psarras et al., 2019). Because fibroblasts are critical for maintaining tissue homeostasis and wound healing through ECM remodelling, there must be a precise balance in the timing and level of their activation. One of the first steps in innate immunity is to activate tissue-resident fibroblasts, at which point they become myofibroblasts. The early presence of myofibroblasts can decrease the severity of myocarditis as they stimulate wound healing. However, if fibroblasts remain active for too long they can cause tissue fibrosis and pathological scarring. This is especially detrimental to organs like the heart where fibrosis inhibits proper muscle function and can lead to heart failure (Souders et al., 2009; Amoah et al., 2015; Vagnozzi et al., 2019).

High levels of complement proteins, PMNs, clotting factors and edema fluid at the site of infection or injury are characteristic of acute inflammation. If the wound can be healed or the pathogen can be cleared, the innate immune response is resolved and either tissue regeneration or fibrotic scarring restores structural integrity to the site of inflammation. If resolution is not possible, a transition from acute to chronic inflammation takes place (Serhan et al., 2010).

### 2.3 Adaptive immunity

The adaptive immune response can take up to seven days to fully develop, hence the need for innate immunity to keep the infection in check initially. It consists primarily of a T-cell mediated and a B-cell mediated pathway (Mahlbacher et al., 2019). Upon activation, B cells differentiate into either plasma cells, which secrete high amounts of antibodies, or memory cells, which are long-lived cells that can quickly produce large amounts of pathogen-specific antibodies if an infection returns (Duchosal, 1997). Myocarditis is a T-cell mediated disease and these cells are essential for its development and progression, but B cells are mostly absent in inflamed cardiac tissue (Chow et al., 1989; Afanasyeva et al., 2004; Meng et al., 2016). Below we will discuss antigen-presenting cells (APCs) and different types of T cells.

#### 2.3.1 Antigen-presenting cells

APCs, including DCs, monocytes and macrophages, are essential in connecting the innate and adaptive immune responses (Gaudino and Kumar, 2019). APCs patrol the body picking up pathogens, related antigens and cell debris on the way, or encounter them in their resident tissue (Hampton and Chtanova, 2019). They use both cell surface pattern recognition receptors, and intracellular receptors to recognize pathogens. Once they have ingested a foreign pathogen or other antigen, they break down the proteins and present them on major histocompatibility (MHC) complexes on their cell surface, ready to prime naïve T cells, which are mature but inactive (Pan et al., 2004; Serhan et al., 2010; Luckheeram et al., 2012; Guerriero, 2019). Circulating DCs are immature, a state characterized by high phagocytic ability and low expression of costimulatory surface proteins (Trombetta et al., 2003; Mbongue et al., 2017; Kim and Kim, 2019). Once they have picked up an antigen, they mature and drain to the lymph nodes.

Maturing DCs stop their uptake of antigens and specialize in antigen presentation and T cell interactions instead (Pan et al., 2004; Mbongue et al., 2017). An important part of this process is an increase in lysosomal function (Trombetta et al., 2003). Immature DCs are inefficient in the way they take up and process antigens. Maturation includes the activation of a proton pump which acidifies the lysozome, aiding proteolysis and efficient formation of peptide-MHC II complexes (Trombetta et al., 2003). Maturing DCs further experience a large increase in synthesis of cytokines and co-stimulatory molecules. One of the cytokines that maturing DCs excrete is interferon (IFN) *γ*, which stimulates the maturation process in an autocrine fashion (Pan et al., 2004). Other cytokines will function as “third signals” once a DC starts interacting with T cells and steering their differentiation (Błyszczuk, 2019). Co-stimulatory proteins like CD40, CD80 and CD86 are transported to the surface of the DC and will likewise play an important role in T cell interactions. Other functional and phenotypical changes include the formation of dendrites, reorganization of the cytoskeleton and the expression of adhesive molecules for migration (Mbongue et al., 2017).

APCs need to drain from the periphery where they have picked up the antigen, to the secondary lymph tissue or spleen (Pan et al., 2004; Mbongue et al., 2017; Hampton and Chtanova, 2019). Once there, they migrate to the T cell zone of the lymph node where they interact with approximately 500–5000 T cells per hour. The minimum number of DCs per lymph node required to elicit a T cell response is estimated to be around 100. An immune stimulus that does not activate enough APCs to meet this threshold will thus not elicit a T cell response (Celli et al., 2012; Obst, 2015; Huang et al., 2019).

#### 2.3.2 T cells

##### 2.3.2.1 Development

T cell progenitors originate in the bone marrow and migrate to the thymus, where they mature (Kumar et al., 2018). During this process, random genetic rearrangements give each cell a unique set of *α* and *β* chains in their TCR, specifying a unique target for them to recognize and respond to. Approximately 10^8^ different recombinant TCRs can be generated in this way (Kwok et al., 2012). Because T cells need to be prevented from recognizing the body’s own proteins and causing an autoimmune reaction, any T cells with TCRs that recognize self-antigens are deleted through negative selection (Serhan et al., 2010; Nurieva et al., 2011; Sikora, 2015). In order for a protein to be recognized as “self” and not elicit an immune response, it must therefore be presented in the thymus (Lv et al., 2011; Bracamonte-Baran and Cihakova, 2017). As MyHC*α* is only present at low levels, however, T cells with TCRs specific to this antigen are present in the body (Rock and Kono, 2008; Lv et al., 2011).

Additionally, T cells with TCRs that have too low or too high an affinity for peptide-MHC complexes are selected against, leaving only cells with mid-range affinities to differentiate into CD4^+^ and CD8^+^ T cells, primarily (Luckheeram et al., 2012). CD4^+^ and CD8^+^ refer to the specific cell surface receptors present. When activated, CD4^+^ and CD8^+^ T cells have distinct phenotypic fates, with CD4^+^ T cells becoming largely Th and Treg cells and CD8^+^ T cells becoming cytotoxic T cells. Thymic output of T cells is mainly important during the first couple of decades of a person’s life and ceases completely after 40–50 years (Kumar et al., 2018). Because the production of naïve T cells is a combination of thymic output and peripheral proliferation, it is difficult to estimate exactly how many new cells are produced by the thymus each day but an upper limit is considered to be around 10^8^ T cells (both CD4^+^ and CD8^+^) per day in humans (Vrisekoop et al., 2008).

All these checks and balances result in a tightly regulated pool of approximately 10^11^ naïve CD4^+^ T cells, with on average 10^5^ cells for each particular epitope–the specific part of the antigen that the T cell recognizes (Kwok et al., 2012; Moro-García et al., 2018). Exactly how many epitope-specific naïve T cells are in circulation depends on the frequency with which the epitope in question is encountered (Kwok et al., 2012). The general size and diversity of the pool of circulating T cells is regulated by a number of soluble factors, most importantly IL-7 (Moro-García et al., 2018).

##### 2.3.2.2 Activation

Mature, naïve T cells leave the thymus and circulate through the body, where they will survive for five to 10 years (Kumar et al., 2018). When they pass through the lymph nodes, they remain there for about half a day before going back into circulation. While in the lymph nodes they scan approximately 160–200 APCs per hour (Obst, 2015). In order for a T cell to be activated, its TCR must recognize the antigen and one of the cell’s co-stimulatory receptors, most importantly CD28, must simultaneously bind its ligand, e.g., CD80 or CD86, which are upregulated on the surface of activated APCs (Hugues, 2010; Serhan et al., 2010; Luckheeram et al., 2012; Guerriero, 2019). Together, these two signals induce activation, clonal expansion and differentiation of T cells. If a T cell is stimulated by an antigen but does not bind any co-stimulatory molecules it will go into anergy, a state in which the cell is hyporesponsive (Wherry, 2011).

In addition to negative selection in the thymus, activation of T cells is an important immune checkpoint and plays a significant role in tolerance of self-antigens. Negative co-stimulatory factors such as CTLA4 and PD-1 are essential immune regulators that prevent the activation of self-specific T cells, and instead induce anergy. Anergic T cells are tolerant towards the self-antigen in question, and are characterized by their inability to proliferate or produce cytokines (Abbas et al., 2004; Nurieva et al., 2011).

A T cell that recognizes the antigen presented by an APC has to stay in contact with that APC for approximately one day to be activated (Obst, 2015). Clonal expansion then takes another three to five days before the activated T cells can differentiate and go back into circulation to attack the source of the antigen (Janeway et al., 2001).

During clonal expansion CD4^+^ and CD8^+^ T cells divide on average seven and 15 times, respectively (Obst, 2015). The daughter cells that an activated T cell produces during clonal expansion will differentiate based on soluble mediators (Hugues, 2010; Obst, 2015). These “third signals” (in addition to the antigen, the “first” signal, and co-stimulatory surface proteins, the “second” signal) consist mainly of cytokines produced by APCs and innate immune cells (Hugues, 2010; Luckheeram et al., 2012; Pennock et al., 2013). Once differentiated, T cells can produce additional cytokines that create positive feedback loops, marginally affecting differentiation (Luckheeram et al., 2012). All together, these cytokines are essential for the development of pro-inflammatory T cells (Błyszczuk, 2019).

##### 2.3.2.3 The effects of ICIs on T cell activation

The immune checkpoint inhibitors ipilimumab and nivolumab both target surface proteins involved in the regulation of T cell activation (Heinzerling et al., 2016). Nivolumab is antibody against PD-1, which is found on the surface of T cells, particularly Th cells (Salmaninejad et al., 2019). T cell activity is inhibited when PD-1 binds one of its ligands, PD-L1 and PD-L2. PD-L1 is expressed on the surface of peripheral cells, such as cardiomyocytes, in response to pro-inflammatory cytokines. PD-L2 is found on the surface of DCs in response to antigen uptake, and the binding of PD-L2 to PD-1 protects the DC against the cytotoxic effects of the activated T cell. It also prevents overactivation of T cells (Lichtman, 2013; Heinzerling et al., 2016; Bracamonte-Baran and Cihakova, 2017; Lu et al., 2019). Binding of PD-1 to either of its ligands induces anergy in the T cell. Blocking PD-1, such as with nivolumab, leads to the stimulation of proliferation, activation and effector functions in CD4^+^ T cells, thus increasing anti-tumour T cell activity. However, it also increases the risk of off-target inflammation in other organs, such as the heart in the case of autoimmune myocarditis (Klein et al., 2018).

Ipilimumab is an antibody targeting CTLA4, which is present on the cell surface of activated T cells. Treg cells in particular constitutively express high levels of CTLA4 (Schmidt et al., 2012; Lichtman, 2013; Heinzerling et al., 2016; Bracamonte-Baran and Cihakova, 2017; Postow et al., 2018). CTLA4 binds to CD80 and CD86 with a higher affinity than CD28, which inhibits T cell activation in a number of ways. First, naïve T cells must compete directly with Treg cells for the binding of CD80 and CD86 on DCs (Pennock et al., 2013). Second, and most importantly, Treg cells remove CD80 and CD86 from DCs through the process of CTLA4-mediated transendocytosis (Ovcinnikovs et al., 2019). Furthermore, the binding of CTLA4 to CD28 stimulates the production of molecules that make the DC toxic to nearby T cells (Sakaguchi et al., 2009). Lastly, CTLA4 inhibits the activation of naïve T cells by stimulating intracellular phosphatase activity which, in turn, inhibits downstream signaling of TCRs and CD28. CTLA4 thus interferes with the functioning of CD28 and the activation of T cells in two ways: directly inhibiting the activity of the DC carrying CD28; and indirectly inhibiting the activity of T cells through DCs (Rudd et al., 2009; Pennock et al., 2013). Treatment with ipilimumab inhibits these regulatory mechanisms and thus leads to a significant increase in T cell activity, both against the tumour and potentially against other organs, causing side-effects such as autoimmune myocardits.

##### 2.3.2.4 Differentiation

The differentiation of CD4^+^ T cells is crucially dependent on the cytokines present. IFN*γ* and IL-12 stimulate differentiation towards the Th1 phenotype (Serhan et al., 2010). IL-12 is largely produced by DCs, and induces production of IFN*γ* in natural killer cells (Luckheeram et al., 2012). IL-2 and IL-4 stimulate differentiation towards the Th2 phenotype. Furthermore, IL-6 plays a dual role in Th2 differentiation as it both stimulates differentiation towards a Th2 phenotype through IL-4 dependent mechanisms, and inhibits differentiation towards a Th1 phenotype by interfering with signaling downstream of IFN*γ* (Luckheeram et al., 2012). IL-6, IL-21, IL-23 and transforming growth factor (TGF)-*β* stimulate the differentiation of Th17 cells in three stages (Sanjabi et al., 2009; Serhan et al., 2010; Zhu et al., 2010; Luckheeram et al., 2012). The first stage is coordinated by IL-6 and TGF-*β* and consists of differentiation. It is important that TGF-*β* is present at low levels and that IL-6 is present simultaneously, otherwise differentiation is diverted towards a regulatory phenotype. The second stage entails self-amplification and is regulated by IL-21, followed by a stabilization stage which involves APC-produced IL-23 (Luckheeram et al., 2012). Differentiation towards an induced Treg (iTreg) phenotype requires high levels of TGF-*β* and the presence of IL-2 (Sanjabi et al., 2009; Serhan et al., 2010; Josefowicz et al., 2012; Luckheeram et al., 2012). The main sources of TGF-*β* are macrophages and T cells (Cheng and Loscalzo, 2017). IL-2 is mainly produced by activated CD4^+^ T cells, although naïve CD8^+^ T cells, DCs and cells in the thymus also contribute (Nelson, 2004).

##### 2.3.2.5 Function: T helper cells

**FIGURE 2:**
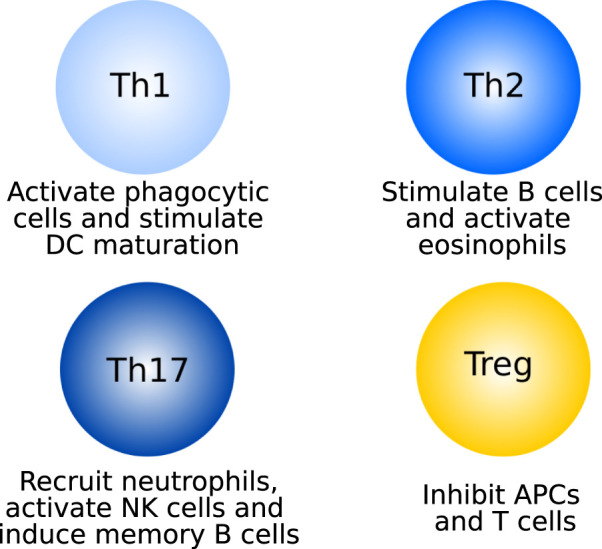
Main functions of the four CD4^+^ T cell subtypes.

Once differentiated, each of the T cell species has a distinct role (see Figure 2 for an overview of CD4^+^ T cells). Th1 cells mainly produce IFN*γ*, through which they activate macrophages and other phagocytic cells, self-amplify differentiation towards the Th1 phenotype, and support DC maturation (Thäle and Kiderlen, 2005; Barin et al., 2010; Zhu et al., 2010; Luckheeram et al., 2012). Th17 cells activate and recruit neutrophils to the site of inflammation. They do this through their main cytokine, IL-17 (Serhan et al., 2010). The self-amplifying cytokine of Th17 cells is IL-21, which furthermore activates NK cells and induces memory functions in B cells (Luckheeram et al., 2012). In the context of myocarditis, the activation of Th1 and Th17 cells against cardiac self-antigens leads to the development of giant cell myocarditis, a rapidly progressing subtype of the disease characterized by the presence of multinucleated giant cells (Kandolin et al., 2013; DeFilippis et al., 2015). Th17 cells and IL-17 have been shown to be dispensable in the early stages of inflammation, but are the main drivers towards dilated cardiomyopathy (Barin and Cihakiva, 2013; Wu et al., 2014; Bracamonte-Baran and Cihakova, 2017).

Th2 cells excrete a variety of cytokines including IL-4, which stimulates the differentiation of more Th2 cells, inhibits differentiation towards the Th1 phenotype and, together with IL-9, stimulates the differentiation and activation of B cells to plasma cells, mediating the antibody response. IL-9 is also involved in the activation of eosinophils, together with IL-5. In addition to these two main functions (stimulating B cells and eosinophils), Th2-secreted cytokines stimulate the differentiation of macrophages towards the M2 phenotype, activate other PMNs and inhibit Th1 cells (Zhu et al., 2010; Luckheeram et al., 2012; Yang et al., 2017). Cardiac antigen-specific Th2 cells, through recruitment of eosinophils, drive the development of eosinophilic myocarditis, one of the most severe forms of the disease (Barin and Cihakiva, 2013; Ong et al., 2015; Ong et al., 2017).

In addition to the heart-specific Th cells whose roles are discussed above, there is a separate function for heart non-specific CD4^+^ effector T cells, such as Th cells. These cells accumulate throughout disease progression and become the major infiltrating subset at a later stage, after the peak of inflammation has passed. Their function lies in protecting the heart from post-inflammatory fibrotic remodelling, thus decreasing the risk of severe negative outcomes such as dilated cardiomyopathy and cardiac dysfunction, which are further discussed in Section 2.4 (Zarak-Crnkovic et al., 2020).

##### 2.3.2.6 Function: T regulatory cells

Treg cells are anti-inflammatory cells, performing key regulatory functions. Whatever the causative factor, it is critical that the immune response is properly regulated as excessive activation of cytotoxic cells could lead to further tissue damage through, for example, leakage of toxic compounds from immune cells (Rock and Kono, 2008; Serhan et al., 2010; Yang et al., 2015). Treg cells inhibit T cells through direct T cell-T cell interactions, through soluble factors and through APCs (Serhan et al., 2010; Schmidt et al., 2012). There are two types of Treg cells: natural Treg cells (nTreg), which develop as Treg cells in the thymus as a separate lineage to other T cells, and iTreg cells, which differentiate as described above. nTreg cells are generally specific to self-antigens, whereas iTreg cells mediate the immune response against non-self-antigens (Luckheeram et al., 2012; Abdel-Gadir et al., 2018). Treg cells inhibit effector T cells, and DCs in a number of ways. The CTLA-4 mediated inhibition of T cell activation has been discussed above. Furthermore, Treg cells interfere with T cell development during proliferation and differentiation, and inhibit the secretion of cytokines by fully developed cells (Sojka et al., 2008; Schmidt et al., 2012). Soluble mediators like IL-10 and TGF*β* directly inhibit effector T cells, primarily by decreasing proliferation and cytokine release (Fiorentino et al., 1991; Rubtsov and Rudensky, 2007; Couper et al., 2008). Other molecules, including fibrinogen-like protein-2, granzyme A/B and adenosine, induce apoptosis in effector T cells, affect their secretion profiles and/or inhibit the maturation of DCs (Lappas et al., 2005; Peterson, 2012). Direct cell-cell contact between Treg cells and Th cells or DCs induces apoptosis in the former and reduced antigen presentation, inhibited maturation and decreased co-stimulation in the latter. Lastly, Treg cells can inhibit Th cells through direct, local competition for growth factors like IL-2, and by competition for space on DCs (Sakaguchi et al., 2009; Peterson, 2012). The level of Treg cells is inversely correlated with the intensity of inflammation in autoimmune myocarditis (Meng et al., 2016).

As previously mentioned, Treg cells constitutively express CTLA4 on their cell surface, making them a target of ipilimumab. In addition to increasing T cell activation by providing a steric block preventing CTLA4 from binding CD28, it has been proposed that another effect of ipilimumab is the depletion of Treg cells all together (Tang et al., 2018; Wei et al., 2018). Although there is no definitive evidence for this, the loss of Treg cells would lead to a significant decrease in inhibition of inflammation, increasing the risk of diseases such as autoimmune myocarditis.

### 2.4 Disease outcomes

Myocarditis has been described as a three-phase disease. The first phase is myocardial injury due to infection or other causes. The second phase consists of autoimmune-mediated damage (Mason, 2003). At this point myocarditis can resolve spontaneously, but in susceptible individuals it can progress to worse conditions (Lichtman, 2013). This is phase three: dilated cardiomyopathy (Mason, 2003). Approximately 9–16% of myocarditis patients progress to this phase, which can lead to heart failure and ultimately death (Wu et al., 2014). Although the phases are described as a chronological progression, there is likely overlap between them and inflammation can be ongoing while dilated cardiomyopathy has occurred (Mason, 2003). The median time between the first dose of ICIs and the onset of myocarditis is approximately 30 days, although some patients present as quickly as 3 days after their first dose (Palaskas et al., 2020).

T cells, PMNs, macrophages, APCs and fibroblasts thus play their own specific roles in the adaptive immune response. Many details have been left out here for the sake of providing an overview, but even so the complexity of the system is apparent. To better understand the pathology of myocarditis, it is necessary to determine which cell types, cytokines and interactions, soluble or contact-mediated, are most important. Mathematical modelling can help us make these determinations, because the minimal set of mechanisms that must be included in the model to reproduce the results observed *in vivo* and *in vitro* are likely to also be critical processes in the development of myocarditis in patients.

## 3 Mathematical modeling

The above discussion shows the immense complexity of the immune response. The need to determine which pathways are critical, and which are of secondary importance, in the development of autoimmune myocarditis motivates the development of a mathematical model. In order to test and verify such a model, it can be calibrated to data from patients or *in vitro* experiments. Methods such as sensitivity analysis can then be applied to the model to select the important cell types and pathways. Furthermore, testing different hypotheses, such as the effect of applying certain drugs or exploring the conditions under which different disease phenotypes occur, is cheaper, safer and quicker in a mathematical model as opposed to an experimental setting. Ultimately, a mathematical model of autoimmune myocarditis as a side-effect of ICIs could be useful in the clinic since it can be calibrated to patient-specific data and used to assess the frequency and size of doses of ICIs an individual patient can tolerate without developing myocarditis. This would help increase the safety of ICI therapies.

**FIGURE 3:**
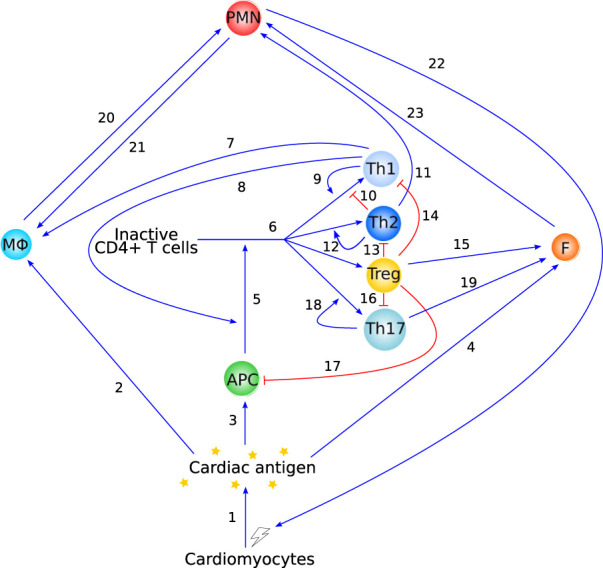
Overview of the immunology behind autoimmune myocarditis as described in Section 2. Blue arrow-heads indicate stimulation, red bar-heads indicate inhibition. Interaction 1 is supported in Section 2.1, interactions 2 and 20 are supported in Section 2.2.1, interactions 3 and 5 are supported in Section 2.3.1, interactions 4 and 23 are supported in Section 2.2.3, interaction 6 is supported in Sections 2.3.2.2 and 2.3.2.4, interactions 7–17 are supported in Section 2.3.2.5, interaction 18 and 19 are supported in Section 2.3.2.6, and interactions 21 and 22 are supported in Section 2.2.2.

As a first step in the development of a mathematical model, we have abstracted the immunology into a diagram showing the main cell types and interactions discussed in Section 2 (see Figure 3). Based on the literature reviewed above, the pathways included in this graph all play a significant role in the development and progression of autoimmune myocarditis and should thus be included, either explicitly or implicitly, in a mathematical model describing this disease. Although Figure 3 focuses on interacting cell types, this does not preclude cytokines from being explicitly included in mathematical models of autoimmune myocarditis. As discussed in Section 2, cytokines play an essential role in mediating the interactions between immune cells and could thus form an important part of a mathematical model. In addition to measurements of cell counts, cytokine levels can then be used to calibrate the model.

We recently published the first mathematical model of autoimmune myocarditis based on a highly simplified version of the graph in Figure 3 (van der Vegt et al., 2022). This model consists of four ordinary differential equations (ODEs) describing the time evolution of dead/damaged cardiomyocytes, innate immune cells, pathogenic CD4^+^ T cells and regulatory T cells, and exhibits steady state behaviour that is consistent with what is observed in patients. In order to keep this first model of autoimmune myocarditis as simple as possible, many cell types and interactions were lumped together. As more data becomes available, the number of dependent variables in this model can be increased to track individual immune cell types and include more details of immunological mechanisms that have been described in this paper. This will further our understanding of the development and progression of autoimmune myocarditis.

We highlight here a number of modelling approaches that describe immunological processes relevant and related to autoimmune myocarditis, and could be used to further develop the theoretical modelling for this disease. We will broadly discuss their context, main results, and how they fit into the schematic in Figure 3. The interested reader is referred to the relevant papers for mathematical detail. We start by reviewing a deterministic, single-scale model consisting of only three ODEs. When modelling a new system for the first time, it is important to start simple to gain a good basic understanding of the system. Simple models can then be extended to include more detail later on. We then discuss models consisting of more complex ODEs, partial differential equations (PDEs) and integro-differential equations to provide inspiration for potential next steps to extend models of autoimmune myocarditis. Much more complicated models, such as stochastic (Fatehi et al., 2018), agent-based (Fachada et al., 2007; Martínez et al., 2012) and multi-scale (Chen et al., 2014) models of (auto)immune reactions, also exist and could be informative in the future. However, given the complexity of autoimmune myocarditis, and the fact that mathematical modelling of autoimmune myocarditis is in its infancy, we do not discuss them here, but refer the reader to the relevant references.

### 3.1 Antigen presentation dynamics

**FIGURE 4:**
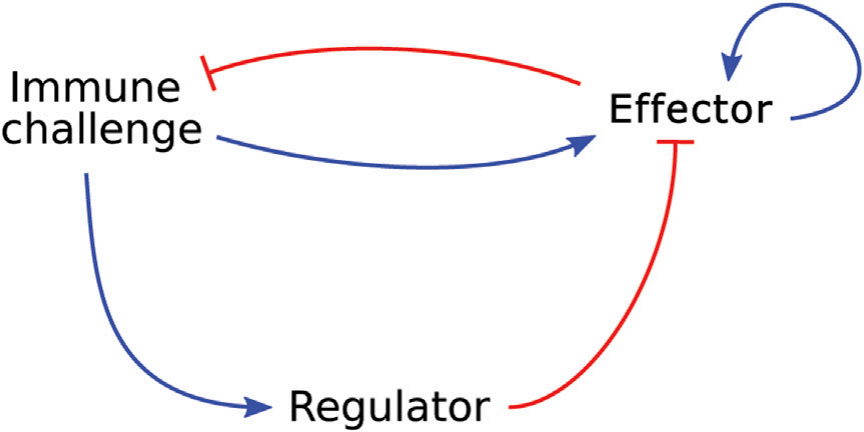
Graphical representation of the model developed by Sontag (2017), which describes a generic immune response in which an effector species and a regulatory species respond to an immune challenge. Blue arrow-heads indicate a stimulating relationship, red bar-heads indicate inhibition.

Sontag (2017) investigated the role of antigen presentation dynamics on the initiation of the immune response. They developed an ODE model to describe the interactions between an immune challenge, such as a tumour or pathogen, an effector species, which can represent Th cells or PMNs for example, and a regulatory species, which can be interpreted biologically as Treg cells. In this model, the immune challenge stimulates both the regulatory and effector species. The regulatory species inhibits the effector species and the effector species stimulates its own proliferation and inhibits the immune challenge (see Figure 4).

Sontag showed that antigen dynamics govern the strength of the immune response. They also show, through a series of simplifications, that their model recapitulates the “two-zone tolerance” that is clinically observed in cancer. In “two-zone tolerance” there are two ranges of per capita growth rate of tumour cells in which the immune system cannot shrink the tumour, and the tumour either grows or remains stable. A growth rate outside these ranges leads to elimination of the tumour. Although the motivation for the model is the study of cancer progression, their model can be applied to other immunological settings as the tumour can easily be replaced by any other pathogen or self-antigen without invalidating the model assumptions. While the model of Sontag (2017) is relevant as a high-level description of the system in Figure 3, it lacks the immunological detail needed to apply it to a specific disease. If the aim is to determine the minimally required set of immune cell types and processes for the development and progression of autoimmune myocarditis, specific cell types need to be included as opposed to generic effector or regulatory species. Furthermore, interactions that are known to happen between effector and regulatory immune cells are missing from this model. For example, Treg cells can inhibit effector T cell proliferation and cytokine production, both of which are not represented in the model by Sontag (2017).

### 3.2 Self-tolerance

**FIGURE 5:**
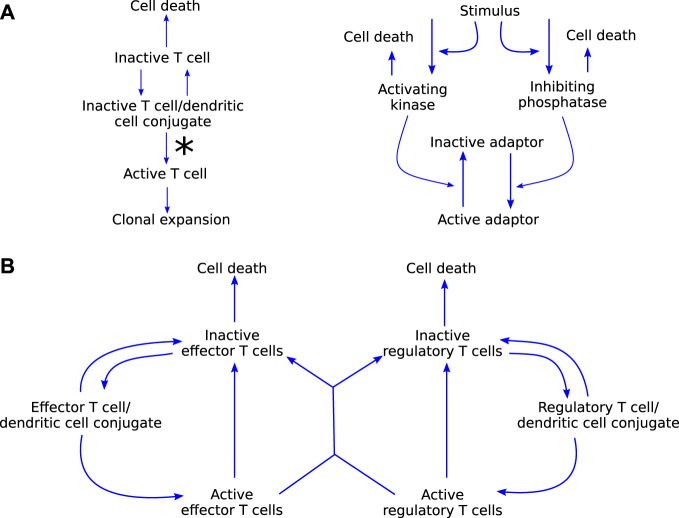
**(A)** graphical representation of model (i) by Carneiro et al. (2005), which explores the hypothesis that self-tolerance is maintained through a T cell activation threshold. The molecular dynamics on the right, specifically the ratio of kinase to phosphatase, influence the rate at which T cells leave the conjugate in an activated state, signified by the asteriks on the left. **(B)** Graphical representation of model (ii) by Carneiro et al. (2005) which explores the hypothesis that regulatory T cells are responsible for maintaining self-tolerance. Blue arrow-heads indicate stimulation.

Carneiro et al. (2005) consider the clonal expansion of T cells, with a focus on immunological self-tolerance. They present two models, adapted from previously published work, to investigate two hypotheses: 1) self-tolerance is maintained through activation thresholds in T cells (Figure 5A); and 2) specific regulatory T cells maintain self-tolerance through inhibition of proliferation of self-reactive effector T cells (Figure 5B). Both of the models are relatively simple in that they consist of only a few ODEs each, but the functional forms within them are complicated, making the system analytically intractable. Through phase plane and bifurcation analyses, Carneiro et al. (2005) find that their model for hypothesis 1) only replicates part of the experimental literature, while their model for hypothesis 2) does not have such limitations. Their main conclusion is that cross-talk between regulatory and effector T cells is sufficient to maintain self-tolerance. They note, however, that mechanisms 1) and 2) are not mutually exclusive and both have been observed in experiments. Because the inhibition of self-tolerance is a critical part of the development of autoimmune myocarditis as a side-effect of ICIs, a model such as this is an excellent starting point for modelling autoimmune myocarditis as one of the most important processes is already well represented mathematically. The immunology included in these models covers primarily interactions 5 and 6 in Figure 3. However, these aspects are only two components of the wealth of interactions in Figure 3. To keep the model of autoimmune myocarditis tractable, each component model needs to be as simple as possible while remaining realistic, and simplifying this model of self-tolerance for the purpose of inclusion in a model for autoimmune myocarditis will thus be essential.

### 3.3 Innate and adaptive immunity

**FIGURE 6:**
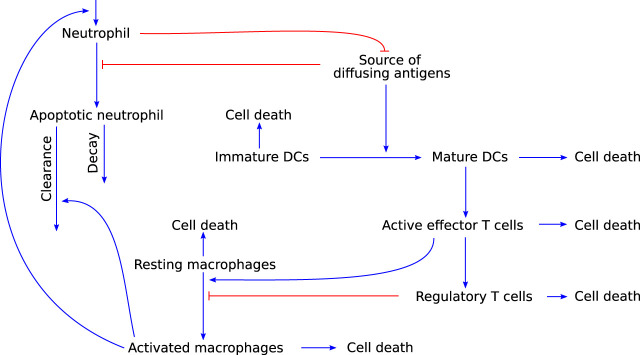
Graphical representation of the model developed by Su et al. (2009), which describes an immune response that includes both the innate and adaptive arm of the immune system. Blue arrow-heads indicate stimulation, red bar-heads indicate inhibition.

Although simple relative to the complexity of the full immune system, the still somewhat complicated PDE model developed by Su et al. (2009) describes the temporal and spatial interactions of various immune cell types and chemokines in response to a source of diffusing antigens (see Figure 6 for details of the non-spatial interactions). The cell types included are active and apoptotic neutrophils, immature and mature DCs, resting and activated macrophages, effector T cells, and regulatory T cells. Generic cytokines and chemokines are used to describe their contact-independent interactions in response to antigens or pathogen-associated molecular patterns. Based on this model, the authors aim to identify a minimally required set of immune system components to describe the immune response. They simulate the model leaving out either Treg cells or DCs and, based on the resulting model dynamics, conclude that all cell types included in their model belong to this minimally required set.

This model is relevant to modelling autoimmune myocarditis because it allows for both the resolution of inflammation as well as persistence of the immune response. This behaviour would be expected of a mathematical model of autoimmune myocarditis as both of these scenarios are known outcomes of the disease. However, some of the model assumptions would need to be adapted for the model to be specific for autoimmune myocarditis. First and foremost, the complexity of the PDEs makes them an undesirable approach for a first model of autoimmune myocarditis. With migration of different cell types between the lymph tissue and the site of inflammation being significant aspects of the adaptive immune response, the spatial context is important in inflammation. However, it adds a level of complexity that precludes the use of analytical approaches to provide initial insights into the model behaviour. It would be preferable to use ODE models, which are much more amenable to analysis, in the first instance. Furthermore, macrophages in this model appear to be only tissue-resident and are not recruited from outside the inflammed tissue, which is an important source of these cell types, as described in Section 2.2.1 (Serhan et al., 2010). Lastly, although this model covers most interactions in Figure 3, cell types such as eosinophils and fibroblasts, and the direct cytotoxic effects of T cells, are not included in this model, but are known to play a role in autoimmune myocarditis. Their addition will therefore be necessary for a biologically realistic model of the disease.

### 3.4 T-cell mediated autoimmunity

**FIGURE 7:**
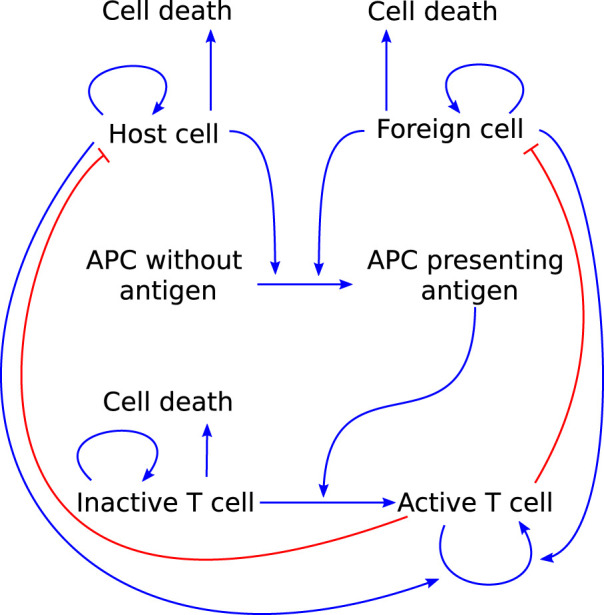
Graphical representation of the model T cell-mediated autoimmunity developed by Delitala et al. (2013) to explore the roles of molecular mimicry, genetic susceptibility and somatic mutations in causing autoimmunity. Arrows pointing out indicate cell death. Blue arrow-heads indicate stimulation, red bar-heads indicate inhibition.

Lastly, we highlight a model that focuses specifically on T-cell mediated autoimmunity. Delitala et al. (2013) use a combination of ODEs and integro-differential equations to model the dynamics of host cells, foreign cells, APCs, and naïve and activated T cells (see Figure 7). They use their model as a virtual laboratory to test the effects of molecular mimicry, genetic susceptibility to autoimmunity, and somatic mutations in the *Fas* gene that cause over-proliferation of T cells and decreased action against foreign cells. Their model can reproduce the effects of molecular mimicry, as seen in experimental work. Furthermore, it suggests that a combination of somatic mutations and molecular mimicry can reproduce the trends seen in patients with autoimmune lymphoproliferative syndrome, indicating that somatic mutations can be a significant trigger for autoimmune disease.

A model of autoimmune myocarditis and the role of ICIs in its development could take inspiration from a model such as this as it already includes a significant portion of the processes involved in the adaptive immune response ((part of) interactions 1, 3, 5, and 6 in Figure 3). Things to consider when adapting this model to describe autoimmune myocarditis are the regulatory roles that T cell phenotypes can play (see Section 2.3.2) and the role of the innate immune response in the development and progression of the disease (see Section 2.2).

These mathematical models, although they do not describe the immunology behind autoimmune myocarditis directly, serve as inspiration for those wishing to further advance the modelling of this disease.

## 4 Conclusion

With the increasing survival rate of many types of cancers comes the need to carefully consider the risks and side effects of cancer treatment, especially as these may have health consequences long after the acute phase (Jain et al., 2017; Peretto et al., 2020). Autoimmune myocarditis, like many other side effects of ICIs, has complex and incompletely understood underlying mechanisms, making it difficult to understand which pathways are most critical. Although it is strongly suggested that both CD4^+^ and CD8^+^ T cells may mediate autoimmune myocarditis with overlapping and differential perspectives, there are many cell types and processes within this pathway, including the exact effects of ICIs, whose significance in the development and progression of disease are poorly determined (Bracamonte-Baran and Cihakova, 2017; Postow et al., 2018; Palaskas et al., 2020). By setting out the biological context and highlighting some relevant mathematical models, we have collected the information necessary to advance the mathematical modelling of autoimmune myocarditis and the effects of ICIs on the development of this disease. With a mathematical model, we can explore and disentangle the immense complexity of the immune system to a set of cell types and interactions that are essential for the development and progression of myocarditis.

The immense complexity of the immune system means there are many unknown factors that can play a role in disease development and should thus be considered for inclusion in the model. These factors include demographics and genetics of specific patients, but also details of the immune system that may currently be unknown. A mathematical model of autoimmune myocarditis will highlight where the gaps in our understanding of the disease mechanisms lie by providing a formal framework in which different hypothesis can be united and tested. It can furthermore suggest experiments that may verify the model by testing its predictions, and provide data for parameter value estimation. This model-predict-refine cycle will be essential in building, verifying and personalising the model in the future.

A minimal model that is able to recapitulate the disease phenotype observed in patients will suggest which processes are likely to be important, help guide and focus experimental efforts, and provide new insights into the mechanisms driving this disease. For example, models will be important for the development of an *in vitro* assay that can test potential new therapeutic avenues for myocarditis-related cardiotoxicity. No such experimental set-up exists to our knowledge, and understanding which cell types are critical and which are of secondary importance will be essential for its development. Furthermore, mathematical models can support experimental work in exploring different treatment regimes through the use of methods such as optimal control theory. They also would provide a quantitative network that integrates relevant risk factors of individual patients to predict the probability of developing autoimmune myocarditis in individual patients. Thus, using mathematical modelling to understand autoimmune myocarditis as a side-effect of ICIs can help in the process of making cancer treatments safer and minimizing their long-term effects.
